# Exploiting biofilm phenotypes for functional characterization of hypothetical genes in *Enterococcus faecalis*

**DOI:** 10.1038/s41522-019-0099-0

**Published:** 2019-09-19

**Authors:** Julia L. E. Willett, Michelle M. Ji, Gary M. Dunny

**Affiliations:** Department of Microbiology and Immunology, University of Minnesota Medical School, Minneapolis, MN USA

**Keywords:** Pathogens, Biofilms, Microbial genetics

## Abstract

*Enterococcus faecalis* is a commensal organism as well as an important nosocomial pathogen, and its infections are typically linked to biofilm formation. Nearly 25% of the *E. faecalis* OG1RF genome encodes hypothetical genes or genes of unknown function. Elucidating their function and how these gene products influence biofilm formation is critical for understanding *E. faecalis* biology. To identify uncharacterized early biofilm determinants, we performed a genetic screen using an arrayed transposon (Tn) library containing ~2000 mutants in hypothetical genes/intergenic regions and identified eight uncharacterized predicted protein-coding genes required for biofilm formation. We demonstrate that OG1RF_10435 encodes a phosphatase that modulates global protein expression and arginine catabolism and propose renaming this gene *bph* (biofilm phosphatase). We present a workflow for combining phenotype-driven experimental and computational evaluation of hypothetical gene products in *E. faecalis*, which can be used to study hypothetical genes required for biofilm formation and other phenotypes of diverse bacteria.

## Introduction

*Enterococcus faecalis* is a ubiquitous Gram-positive microbe found in water, soil, and as a commensal organism in the gastrointestinal (GI) tracts of humans and animals.^1–3^ Enterococci are resistant to many chemical and physical stresses, including pH, temperature, and bile salts.^4,5^ They are prevalent in the neonatal GI tract and are maintained as low-abundance commensals in adults.^6,7^ *E. faecalis* also causes life-threatening opportunistic infections and is a leading cause of nosocomial infections on host tissues such as heart valves and the urinary tract as well as implanted devices like prosthetic joints and catheters.^8–11^ Treatment of *E. faecalis* infections is complicated by intrinsic and acquired antimicrobial resistance.^12–14^

One factor that contributes to *E. faecalis* colonization, infections, and antibiotic resistance is biofilm formation. Biofilms develop in a multi-step process during which bacteria attach to a surface, elaborate a complex matrix, and grow as a surface-associated bacterial community.^15–18^ *E. faecalis* biofilms can develop in vitro on a variety of substrates and in vivo on tissues and implanted devices, causing biofilm-related infections.^19,20^ Approximately 100 genetic determinants of biofilm formation have been identified in *E. faecalis*, including adhesins, pili, proteases, regulators of extracellular DNA (eDNA) release, transcription factors, and autolysin.^16,21–24^ Additional components such as exopolysaccharides are involved in overall biofilm architecture, and play a critical role in the enhanced resistance of biofilm cells to antimicrobial agents.^25,26^

**Fig. 1:**
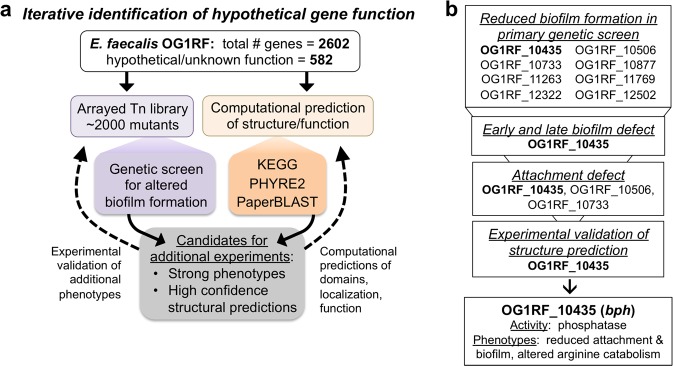
Workflow for characterizing hypothetical gene products important for biofilm formation in *E. faecalis*. **a** An arrayed library of *E. faecalis* OG1RF transposon mutants was constructed using strains with insertions in poorly characterized/hypothetical genes and intergenic regions and was used in a genetic screen to identify biofilm determinants. The protein sequences of low-biofilm gene products were examined to predict structure and function. This data was used to identify top candidate genes for in vitro validation of function. **b** From this workflow, we identified eight genes involved in early biofilm formation, three of which are important for in vitro surface attachment. Further experiments identified OG1RF_10435 (*bph*) as a phosphatase

Nearly a quarter of the genes (582 of 2602) annotated in the *E. faecalis* OG1RF genome (NC_017316) encode products that are annotated as hypothetical (*n* = 445) or proteins containing domains of unknown function (*n* = 137). Many of these uncharacterized loci are involved or expressed during biofilm formation,^22,23^ in animal models of infection,^27,28^ and after exposure to antibiotics,^29^ suggesting that uncharacterized gene products play critical roles in enterococcal ecology and pathogenesis. Our laboratory recently developed an arrayed library of *E. faecalis* OG1RF transposon (Tn) mutants, which includes ~2000 Tn insertions in hypothetical genes and intergenic regions that could encode regulatory RNAs.^23,30^ Here, we used this collection in a genetic screen to identify uncharacterized loci that influence early biofilm formation and combined these results with computational predictions and biochemical analysis to identify gene function of uncharacterized biofilm determinants (Fig. 1).

We found Tn mutations in eight hypothetical genes (OG1RF_10435, OG1RF_10506, OG1RF_10733, OG1RF_10877, OG1RF_11263, OG1RF_11769, OG1RF_12322, and OG1RF_12502) that lead to a defect in early biofilm production and 1 gene (OG1RF_10435) required for both early and late biofilms. We show that OG1RF_10435 is required for surface attachment and that this purified protein has phosphatase activity. Additionally, we provide evidence that deletion of OG1RF_10435 leads to widespread differences in protein expression and alters arginine catabolism. This study demonstrates that genetic screens based on biofilm growth represent a fruitful starting point for characterization of uncharacterized gene products and presents a successful workflow for functional analysis of hypothetical proteins in *E. faecalis*. Similar approaches can likely be applied to the study of uncharacterized loci in other bacteria.

## Results

### Arrayed Tn library screen to identify biofilm defects

Previously, our laboratory developed an arrayed *E. faecalis* OG1RF Tn library consisting of ~15,000 mutants.^23,30^ To facilitate the study of uncharacterized genomic loci, we identified strains in which Tn insertions disrupt hypothetical genes or intergenic regions (*n* ≈ 2000, Supplementary Data 1) and created a smaller library distributed across 22 microtiter (96-well) plates with 1 unique clone per well. When possible, multiple insertions per locus were included in the library. Such redundancy can increase the likelihood of identifying phenotypes for a given region and provide confidence for phenotypes observed during genetic screens.^31^ Using this library, we performed a screen to identify hypothetical genes required for early biofilm production (*t* = 6 h) in TSB without dextrose (TSB-D) growth medium using an established microtiter plate assay.^23,25^ For each mutant, a biofilm index value was calculated as the ratio of biofilm biomass to planktonic cell density relative to parental OG1RF (Supplementary Data 2).

**Fig. 2:**
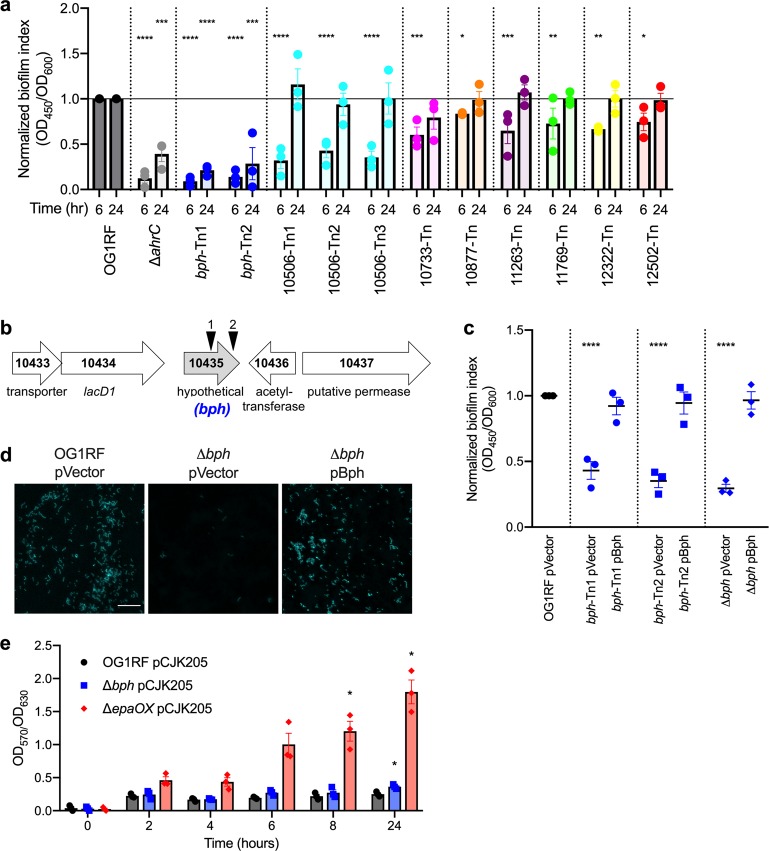
Mutations in *E. faecalis* hypothetical genes lead to decreased biofilm formation. **a** Biofilm production of Tn mutants chosen from arrayed Tn library screen was measured at 6 and 24 h. Values are normalized to biofilm produced by OG1RF (black horizontal line). Statistical significance at each time point was calculated by one-way ANOVA (****q < 0.0001, ***q < 0.001, **q < 0.01, *q < 0.05 with multiple comparisons by two-stage step-up method of Benjamini, Krieger, and Yekutieli). **b** Gene neighborhood of *bph* (gray arrow). Gene numbers indicate “old locus tag” identifiers from the annotated OG1RF genome (NC_017316). Tn insertions are designated by triangles and are numbered based on position in gene relative to the 5’ end (1 = 56.3%, 2 = 91.9%). **c** Biofilm production and complementation in *bph* mutants. Strains carried either an empty vector plasmid (pVector) or complementation plasmid pBph and were cultured in microtiter dishes for 6 h. Values were normalized to parental OG1RF. Statistical significance was calculated by one-way ANOVA as in panel **a**. **d** Fluorescence microscopy of biofilms grown on Aclar substrates for 6 h and stained with Hoechst 33342 (scale bar = 20 μm). Images were obtained with a ×20 objective (×200 total magnification) and are representative of three independent experiments. **e** Cell envelope integrity of Δ*bph*. Strains carried a constitutive *lacZ* expression vector (pCJK205) and were cultured in the presence of chlorophenyl red-*β*-d-galactopyranoside (CPRG). The ratio of CPRG activity (OD_570_) to cell density (OD_630_) was calculated at each time point. Significance was calculated by two-way ANOVA (*q < 0.05 with multiple comparisons as in panel **a**. In panels **a**, **c**, and **e**, each data point represents a biological replicate (*n* = 3). Error bars represent standard error of the mean

In the primary screen, we identified 62 Tn mutants with insertions in hypothetical genes that produced < 25% of the biofilm produced by parental *E. faecalis* OG1RF and 49 Tn mutants with biofilm levels > 125% of OG1RF (Supplementary Fig. 1, Supplementary Data 2). We retrieved 11 presumptive biofilm-defective mutants spanning eight genes and measured in vitro biofilm production at 6 and 24 h (Fig. 2a, Table 1). These mutants had the lowest biofilm index values identified in the primary screen (excluding negative biofilm index values), and none of these genes were previously identified as determinants of biofilm formation.^22,23^ At 6 h, all mutants had reduced biofilm levels (reduction ranging from 17% to 91%) relative to OG1RF (Fig. 2a). The most severe biofilm defects were observed for strains with insertions in OG1RF_10435 and OG1RF_10506, where 6 h biofilm formation was reduced 57–91% relative to OG1RF (Fig. 2a, blue and cyan dots). The OG1RF_10435 Tn mutants also had attenuated biofilm production (~70% reduction relative to OG1RF) at 24 h (Fig. 2a). OG1RF_10435-Tn biofilm levels were comparable to those of the negative control strain Δ*ahrC* (Fig. 2a), which lacks a transcription factor that regulates expression of pili essential for biofilm formation.^24,32^ We also measured 6 h biofilm production of 10 Tn mutants with high biofilm levels in the primary screen, but none had a reproducibly significant increase in biofilm (Supplementary Fig. 1). Therefore, we chose to focus on the role of OG1RF_10435 in biofilm formation for this study, since its disruption resulted in the strongest biofilm defects at both time points. Based on results presented below, we propose designating OG1RF_10435 *bph* (*b*iofilm *ph*osphatase) and henceforth refer to this gene as *bph*.

**Table 1:** Hypothetical genes with low-biofilm phenotypes identified in this study

Locus Tag	NCBI annotation	Length (amino acids)	Pfam domains (KEGG)	Putative structural homologs from Phyre 2 (confidence %, coverage %)
OG1RF_10435	DUF402 domain-containing protein	177	DUF402	Phosphatase SC4828 (100, 90)
OG1RF_10506	hypothetical protein	172	DUF1506	Poly-beta-1,6-n-acetyl-d-glucosamine n-deacetylase (88.6, 66)
OG1RF_10733	YggT family protein	69	YGGT	Ubiquitin-like protein (13.5, 23)
OG1RF_10877	DUF898 family protein	101	DUF898, DUF4234	Conserved domain protein (19.2, 23)
OG1RF_11263	hypothetical protein	344	HTH_40, GerE, DUF1818	“Winged helix” DNA-binding domain (99.6, 27)
OG1RF_11769	DNRLRE domain-containing protein	1259	Big_4, Beta_helix	Lacto-N-bisodase (100, 41)
OG1RF_12322	peptidase C39 family protein	258	Peptidase_C39_2, Peptidase_C70, Sec16	Putative C39-like peptidase (99.9, 53)
OG1RF_12502	WxL domain-containing protein	1563	WxL	Putative peptidoglycan-bound protein (93.1, 5)

*bph* encodes a hypothetical protein 177 amino acids in length that is predicted to localize to the cytoplasm.^33^ It is annotated with a single DUF402 domain (Table 1), and the Tn insertions identified in the biofilm screen are located 56.3% (Tn1) and 91.9% (Tn2) of the total gene length relative to the 5′ end (Fig. 2b). We constructed a markerless in-frame deletion of *bph* (Δ*bph*) and found that it had a biofilm defect comparable to the Tn mutants (Fig. 2c). Expression of *bph* from a pheromone-inducible complementation vector restored biofilm formation in all mutants (Fig. 2c). To visually examine biofilms, we cultured OG1RF and Δ*bph* strains on Aclar discs, stained cells with Hoechst 33342, and observed adherent biofilm cells using fluorescence microscopy. OG1RF biofilm was visible across the surface of the substrate, but very few Δ*bph* cells were present (Fig. 2d, left vs. middle panel). An increase in the number of attached biofilm cells was observed with a Δ*bph* strain expressing *bph* from a plasmid (Fig. 2d, right panel).

One explanation for reduced biofilm formation by *bph* mutants is increased cell lysis due to cell envelope defects. To test this, we evaluated cell integrity using a colorimetric growth assay in which cells constitutively expressing *lacZ* were cultured in the presence of chlorophenyl red-*β*-d-galactopyranoside (CPRG). CPRG is a LacZ substrate that does not cross the cell membrane, so hydrolysis occurs when cell envelope defects allow CPRG to enter cells or when LacZ is released into the growth medium by cell lysis. We measured CPRG hydrolysis (OD_570_) relative to cell density (OD_630_) to determine whether cell envelope defects or lysis contributed to the reduced biofilm of *bph* mutants. In the positive control strain OG1RF Δ*epaOX*, which lacks a glycosyltransferase required for envelope integrity,^25^ CPRG hydrolysis relative to OG1RF increased 5.7-fold after 8 h and 7.16-fold after 24 h (Fig. 2e). Hydrolysis in the Δ*bph* strain was not significantly different than OG1RF until 24 h, at which point a 1.4-fold increase was observed. Therefore, the early biofilm defects observed with *bph* mutants cannot be explained by aberrant cell integrity, and it is unlikely that the modest signal observed at 24 h could explain the entire biofilm-defective phenotype.

### *bph* is required for surface attachment and pili production

**Fig. 3:**
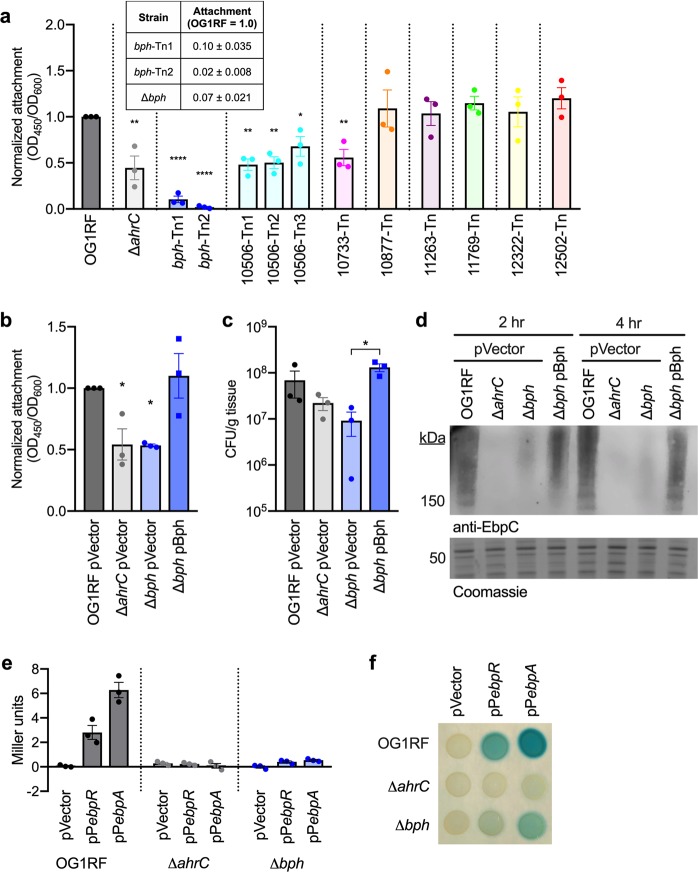
*bph* is required for surface attachment and pili expression. **a** Attachment of Tn mutants to polystyrene microtiter plates was measured after 1 h. Attachment was calculated as the ratio of attached biomass (OD_450_) to cell density (OD_600_) relative to OG1RF. Significance was determined by one-way ANOVA (*q < 0.05 with multiple comparisons by two-stage step-up method of Benjamini, Krieger, and Yekutieli). The inset table shows attachment values (mean of three biological replicates) for the *bph* Tn mutants and clean deletion. **b** Attachment to polystyrene microtiter plates was measured as in panel **a**. Strains carried either an empty vector or the complement plasmid pBph. Δ*ahrC* (light gray) and Δ*bph* (light blue) strains had reduced attachment (significance determined as in panel **a**). Attachment was increased by a plasmid expressing *bph* (dark blue). **c** Strains from panel **b** were cultured with porcine heart valves, and tissue-associated colony forming units were quantified. Δ*ahrC* and *Δbph* strains had reduced average attachment relative to OG1RF, and expressing *bph* from a plasmid resulted in a significant increase in attachment relative to the empty vector strain (significance determined as in panel **a**). **d** Pili protein in whole-cell lysates isolated after 2 or 4 h of growth was detected by Western blotting with an anti-EbpC antibody. **e** Activities of the *ebpR* and *ebpA* promoters were measured using cells carrying a vector control (pTCV-LacSpec) or promoter fusions for the indicated genes. Values represent Miller units calculated from β-galactosidase assays over three biological replicates. **f** The strains used in panel **d** were spotted on plates containing X-gal and imaged. In panels **a**, **b**, **c**, and **e**, each data point represents a biological replicate (*n* = 3). Error bars represent standard error of the mean

Biofilm production is a complex process requiring attachment to a surface and elaboration of an extracellular matrix, and we hypothesized that the low-biofilm phenotypes observed for Tn mutants in this study were due to disruption of discrete steps in the biofilm development pathway. First, we asked whether the 11 Tn mutants tested for biofilm formation could attach to surfaces. We incubated concentrated, washed cells in a microtiter dish for 1 h (a time period sufficient for attachment, but not for extensive growth of adherent bacteria) and stained the attached biomass with safranin. *bph*-Tn mutants had ~90% less attachment than parental OG1RF, and OG1RF_10506-Tn, OG1RF_10733-Tn, and Δ*ahrC* strains had a ~50% reduction in attachment (Fig. 3a). The plasmid-free *bph* deletion mutant phenocopied the attachment defect observed with the Tn mutants (Fig. 3a, inset table), and expression of *bph* from a plasmid in the Δ*bph* mutant restored attachment to the level of parental OG1RF (Fig. 3b).

Next, we asked whether *bph* is required for attachment to a relevant host surface. *E. faecalis* is a common cause of infective endocarditis, during which bacteria attach to heart valve surfaces and form biofilm-like lesions called vegetations.^34,35^ Our lab previously established an ex vivo porcine heart valve model to study virulence factors and endocarditis-associated isolates,^36,37^ and we used that here to evaluate attachment. Relative to OG1RF, a reduction in tissue-attached cells was observed for Δ*ahrC* (3.1-fold) and Δ*bph* (7.6-fold) (Fig. 3c). Expressing *bph* from a plasmid in Δ*bph* resulted in a ~1 log increase (*q* < 0.05) in the number of tissue-associated cells over the empty vector strain (Fig. 3c).

In *E. faecalis*, Ebp pili are well-characterized determinants of surface attachment, biofilm formation, and virulence.^24,38–40^ Because the Δ*bph* mutant had reduced attachment, we asked whether pilus expression was altered in this strain. We isolated whole-cell lysates after 2 or 4 h of growth and detected pilin protein by Western blot using an antibody to the major pilus subunit EbpC.^41^ The high molecular weight banding pattern characteristic of pili subjected to electrophoretic separation is present in the OG1RF lysate (Fig. 3d). As shown previously, lysate from the Δ*ahrC* strain had no detectable pili.^24^ The Δ*bph* lysate had reduced levels of pili relative to OG1RF at both 2 and 4 h, and production of pili was increased in the complemented mutant (Fig. 3d).

Expression of *ebpABC* is regulated by the transcription factor EbpR, and *ebpR* is regulated by itself and AhrC.^24^ Therefore, we hypothesized that decreased pili levels in the Δ*bph* strain could be caused by misregulation of EbpR or the structural pilus genes, and we examined expression from the *ebpR* and *ebpA* promoters using transcriptional *lacZ* fusion constructs. These reporter constructs were previously used to examine regulation of pili genes by *ahrC*.^24^ In liquid culture, pP*ebpR* had ~7-fold higher activity in OG1RF compared to Δ*bph* (2.81 vs. 0.40 Miller units), and pP*ebpA* had ~12-fold higher activity (6.28 vs. 0.54 Miller units) (Fig. 3e). In agreement with previous work,^24^ expression from both reporters was eliminated in the Δ*ahrC* mutant (Fig. 3e). On indicator plates containing X-Gal (5-bromo-4-chloro-3-indolyl-β-d-galactopyranoside), blue pigmentation indicative of *lacZ* expression was observed for OG1RF strains carrying pP*ebpR* and pP*ebpA* (Fig. 3f). No color was observed for Δ*ahrC*. Δ*bph* strains carrying pP*ebpR* and pP*ebpA* had observable blue color that was lighter than the corresponding parental OG1RF strain, which correlates with the reduced expression in liquid culture.

### Effect of *bph* on diverse biofilm determinants and plasmid transfer

Gelatinase, eDNA, polysaccharides, and the collagen-binding surface adhesin Ace contribute to biofilm architecture and virulence, and biofilm-deficient mutants with disruptions in genes encoding these products have been characterized.^15,16,25,26,42,43^ Polysaccharides extracted from OG1RF and Δ*bph* were analyzed using native gel electrophoresis, and there were no gel migration differences between the samples (Supplementary Fig. 2). We compared eDNA from OG1RF and Δ*bph* and found that planktonic Δ*bph* cells produced higher levels of eDNA than OG1RF (Supplementary Fig. 2), suggesting that a lack of eDNA is not responsible for the reduced biofilm phenotype. Additionally, no defect in gelatinase activity was observed for Δ*bph*, as determined by growth on agar plates supplemented with 3% gelatin (Supplementary Fig. 2). AhrC also regulates *ace* in conjunction with ArgR2.^24^ Because Δ*bph* has reduced pilus expression, we asked whether production of Ace is affected in this strain. We harvested total cell lysates after 4 h of growth at 46 ^o^C and performed Western blots using an anti-Ace antibody. Ace levels were unchanged in Δ*bph* relative to OG1RF (Supplementary Fig. 2). A *bph* mutant also produced normal levels of SA80, an abundant OG1RF cell surface antigen (Supplementary Fig. 2).^44^

Pheromone-mediated conjugative plasmid transfer is an important source of horizontal gene transfer for *Enterococcus*, and expression of aggregation substance (PrgB) following induction of the conjugative plasmid pCF10 is involved in cell aggregation and biofilm formation.^45–48^ Therefore, we asked whether a *bph* mutant could participate in plasmid transfer and whether *bph* affected PrgB expression and aggregation. We performed liquid mating experiments with *E. faecalis* OG1Sp pCF10 as a donor and found that pCF10 could be transferred to Δ*bph* recipient cells at a rate similar to OG1RF (Supplementary Fig. 3a). We next asked whether Δ*bph* carrying pCF10 could donate the plasmid to OG1Sp recipients and found only a modest reduction in plasmid transfer per donor cell after 1 h (3.3-fold reduction) and 4 h (2.1-fold reduction) of co-culture relative to OG1RF pCF10 (Supplementary Fig. 3b). To detect PrgB, we induced OG1RF pCF10 and Δ*bph* pCF10 cultures with exogenous cCF10 pheromone (10 ng/mL), isolated protein after 1 h, and probed for PrgB using a Western blot. Numerous studies examining PrgB by Western blot have revealed full-length (~150 kDa) and processed (~78 kDa) proteins,^49,50^ both of which were observed in lysate from OG1RF pCF10 (Supplementary Fig. 3). The *bph* mutant had reduced levels of both PrgB species. This suggests that the modest decrease in plasmid transfer efficiency for Δ*bph* donor cells could be due to reduced PrgB levels. Δ*bph* pCF10 cells aggregated at the same rate as OG1RF pCF10 following addition of exogenous cCF10 (Supplementary Fig. 3d). Taken together, these data suggest that *bph* is required for *E. faecalis* surface attachment and is involved in Ebp pilus regulation, but that it is not required to produce other biofilm determinants or surface proteins in general.

### Deletion of *bph* leads to global changes in protein expression

**Fig. 4:**
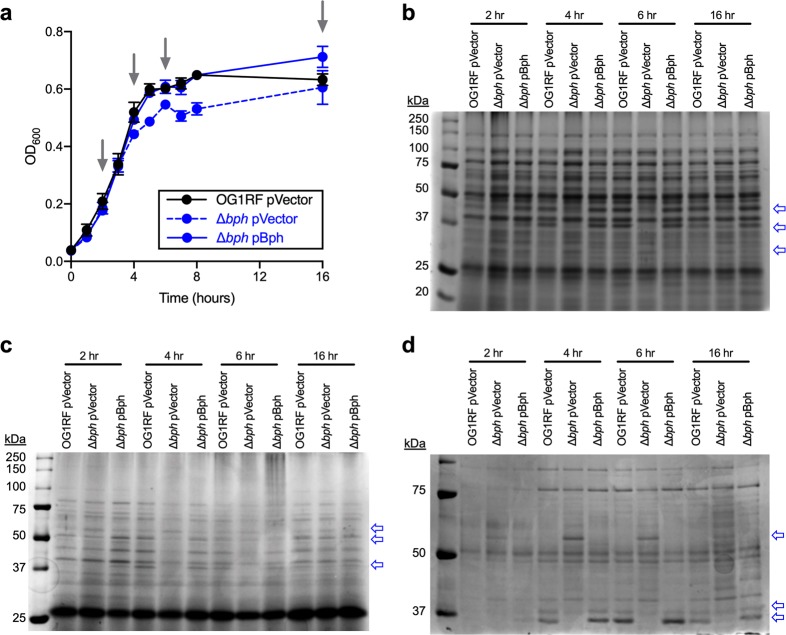
Disrupting *bph* results in temporal changes in global protein expression. **a** Growth curves of the indicated strains in TSB-D growth medium. Data points represent the mean of three biological replicates. Error bars represent standard error of the mean. Aliquots were removed for protein analysis at time points indicated by arrows. Whole-cell lysates were separated into **b** protoplast and **c** cell wall fractions using a lysozyme treatment. **d** Secreted proteins were precipitated from the supernatant with trichloroacetic acid. All protein samples were subjected to SDS–PAGE and stained with Coomassie. Blue arrows to the right of gel images denote protein bands that are differentially expressed in Δ*bph*

Surface attachment and biofilm formation are complex processes that require coordination of myriad gene products. Therefore, we asked whether we could observe qualitative differences in global protein production between OG1RF and Δ*bph* using SDS–PAGE. We grew OG1RF and Δ*bph* carrying an empty vector or the pBph complementation construct and measured growth as the optical density at 600 nm (OD_600_) (Fig. 4a). The Δ*bph* mutant has a slight growth defect relative to OG1RF in early stationary phase (Fig. 4a, compare black and dashed blue lines) that is complemented by expression of pBph (Fig. 4a, compare dashed and solid blue lines). At indicated time points (Fig. 4a, gray arrows), we isolated proteins from protoplasts, the cell wall, and culture supernatants and analyzed these using SDS–PAGE and Coomassie staining. Distinct differences were apparent in the protein banding patterns of OG1RF and Δ*bph* (Fig. 4b–d). Several proteins were missing in the vector control Δ*bph* strain compared to OG1RF (Fig. 4b, top two arrows), and others were present in Δ*bph* but absent in OG1RF (Fig. 4d, top arrow). We also observed temporal differences in differential protein expression. Banding patterns between OG1RF and Δ*bph* at *t* = 2 h were more similar in all cellular fractions than at *t* = 4 h and *t* = 6 h. Protein patterns of the Δ*bph* strain expressing *bph* from a plasmid matched those of OG1RF.

**Fig. 5:**
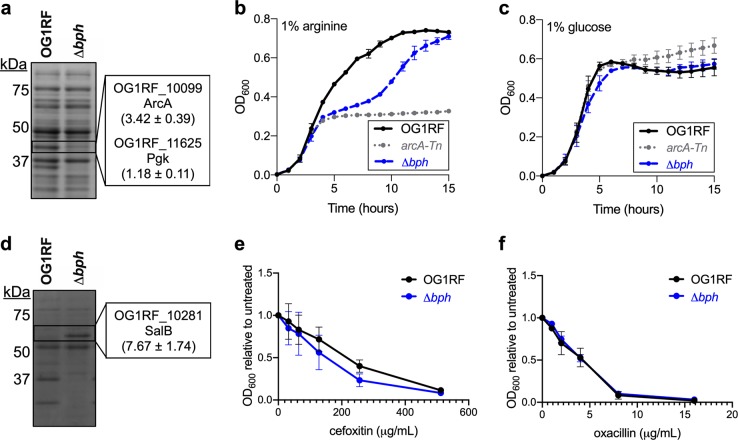
Effect of deleting *bph* on arginine catabolism and response to antibiotics. **a** SDS–PAGE image showing an abundant protein band (boxed region) present in lysate from OG1RF but not Δ*bph*. Gel slices corresponding to the boxed areas were excised and analyzed via LC–MS/MS. Ratios indicate values in total normalized spectral counts. **b** Growth of parental OG1RF (black line), the *arcA* null strain *arcA*-Tn (gray line), and Δ*bph* (blue line) in semi-defined medium supplemented with 1% arginine. Growth was measured by absorbance at 600 nm (OD_600_). *arcA*-Tn has reduced growth relative to OG1RF, and Δ*bphA* has a diauxic growth pattern. **c** The strains from panel **b** were grown in semi-defined medium supplemented with 1% glucose. All strains have similar growth patterns. **d** SDS–PAGE image showing an abundant protein band (boxed region) present in Δ*bphA* but not OG1RF. Gel slices were analyzed as in panel **a**. OG1RF and Δ*bph* were grown in varying concentrations of **e** cefoxitin and **f** oxacillin. The final OD_600_ value at each antibiotic concentration was divided by the OD_600_ of an untreated culture. For panels **b**, **c**, **e**, and **f**, each data point represents the mean of three biological replicates. Error bars represent standard error of the mean.

We excised gel slices corresponding to two protein bands in the 4 h samples and used liquid chromatography–tandem mass spectrometry (LC/MS–MS) to identify peptides (Fig. 5). The band between 37 and 50 kDa in the protoplast fraction was present in OG1RF but reduced in Δ*bph* (Fig. 5a). LC/MS–MS revealed that peptides mapping to two proteins, ArcA (arginine deiminase) and Pgk (phosphoglycerate kinase), were more abundant in OG1RF (3.42 and 1.18 times higher, respectively) than Δ*bph*. The band between 50 and 75 kDa in the secreted protein fraction was present in Δ*bph* but not in OG1RF (Fig. 5d). Peptides mapping to SalB were ~7.5 times higher in Δ*bph* than OG1RF, suggesting that more SalB accumulates in the supernatant of Δ*bph*-mutant cultures than OG1RF.

Both proteins that were more abundant in OG1RF than Δ*bph* are linked to metabolism. *E. faecalis* can use arginine as an energy source, and ArcA converts l-arginine to l-citrulline as the first step in the arginine catabolic pathway.^51,52^ Pgk generates ATP during glycolysis.^53^ Reduced levels of these proteins in Δ*bph* led us to hypothesize that this strain would have a growth defect relative to OG1RF in arginine or glucose. To test this, we compared the growth of OG1RF, Δ*bph*, and an *arcA*-null strain (*arcA*-Tn) in a semisynthetic medium supplemented with arginine or glucose^52^. In medium supplemented with arginine, all strains had similar growth for the first 3 h, at which point the *arcA*-Tn mutant and Δ*bph* mutant appear to enter stationary phase (Fig. 5b, black vs. gray line). Interestingly, Δ*bph* exhibits diauxic growth in the presence of arginine (Fig. 5b, blue line). All strains had similar growth in medium supplemented with glucose (Fig. 5c), suggesting that the diauxic growth of Δ*bph* in arginine is not due to a general growth defect. Together, the mass spectrometry and growth experiments provide evidence that Bph affects arginine utilization via the arginine deiminase pathway.

LC/MS–MS showed that SalB is present at higher levels in the supernatant of Δ*bph* compared to OG1RF (Fig. 5d). SalB is a putative peptidoglycan hydrolase that contributes to cephalosporin resistance in *E. faecalis*.^54^ Therefore, we hypothesized that increased levels of SalB in Δ*bph* would lead to increased resistance against cell wall-active antibiotics. We grew OG1RF and Δ*bph* in TSB-D supplemented with varying concentrations of cefoxitin (Fig. 5e) and oxacillin (Fig. 5f) and measured the ratio of final OD_600_ values for treated cultures relative to an untreated culture. Surprisingly, we observed a slight shift towards sensitivity for Δ*bph* in cefoxitin (Fig. 5e) and no difference between OG1RF and Δ*bph* in oxacillin (Fig. 5f), suggesting that the increased levels of SalB detected in the Δ*bph* supernatant do not lead to a general increase in tolerance to these antibiotics.

### Purified Bph has phosphatase activity

**Fig. 6:**
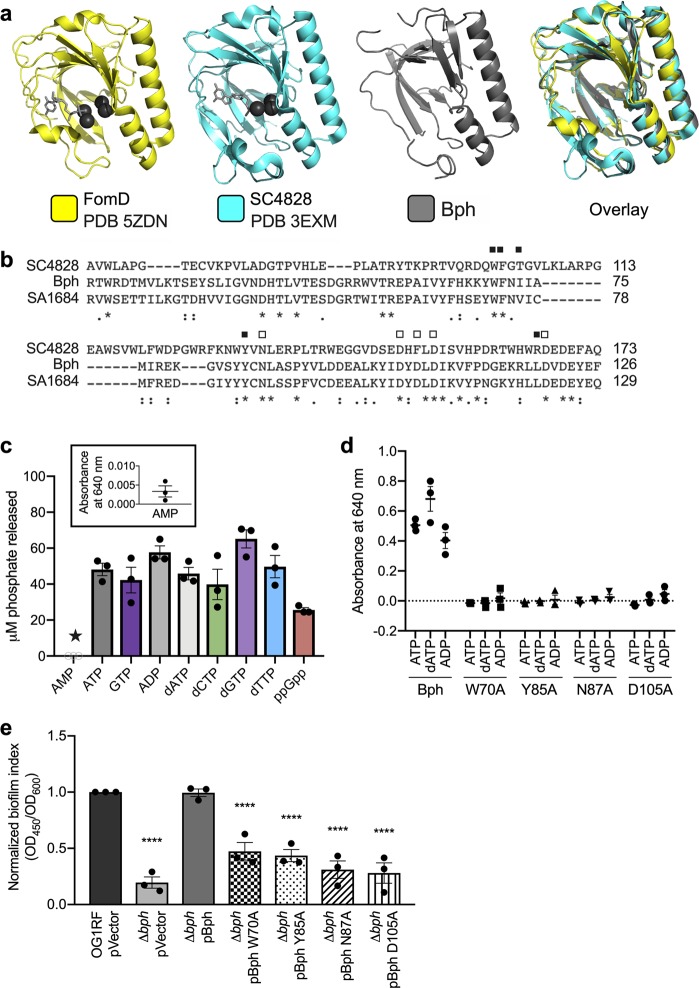
Bph has predicted structural homology to phosphatases and has phosphatase activity in vitro. **a** Predicted structural homologs identified by Phyre2. The structure of SC4828 (cyan, PDB 3EXM) was used to model Bph (gray). The overlay was generated from crystal structures of FomD (yellow, PDB 5ZDN) and SC4828 and the predicted structure of Bph. Substrates are shown as light gray sticks (CDP for FomD, GPCP for SC4828), and coordinated divalent cations are shown as dark gray spheres (Mg^2+^ for FomD, Ca^2+^ for SC4828). **b** Alignment (Clustal2 Omega) of active site residues in SC4828, SA1684, and Bph. Asterisks and dots (bottom) indicated amino acid relatedness. Filled squares (top) signify ligand-binding residues and open squares (top) signify residues that coordinate Ca^2+^ in SC4828. Numbers (right) indicate amino acid positions. The Tn insertions in Bph occur at Y101 (Tn1) and outside of the portion of the alignment shown (Tn2). **c** Purified wild-type Bph-H6 or **d** point mutant variants were mixed with the indicated substrates. A malachite green-based assay was used to quantify phosphatase activity. Activity on AMP (marked with a star) was below the detectable limit, so *A*_640_ values are shown (inset, panel **c**). **e** Biofilm production at 6 h was determined for Δ*bph* cells carrying *bph* complementation plasmids with the inactive point mutants from panel **d**. Significance was determined by one-way ANOVA (****q < 0.0001 with multiple comparisons by two-stage step-up method of Benjamini, Krieger, and Yekutieli). In panels **c**–**e**, each data point represents a biological replicate (*n* = 3). Error bars represent standard error of the mean

Because *bph* is annotated as a hypothetical gene, we asked whether we could glean clues about gene product function from computational predictions. We used the sequence of Bph as an input for Phyre2^55^ and identified two high-confidence predictions to known crystal structures in the Protein Data Bank (PDB) (Fig. 6a). The predicted alignments to FomD (PDB 5ZDN) and SC4828 (PDB 3EXM), span > 90% of the Bph protein sequence, despite low sequence homology (17% between Bph/SC4828 and 14% between Bph/FomD). FomD hydrolyzes cytidylyl (*S*)-2-hydroxypropylphosphonate during fosfomycin biosynthesis in *Streptomyces*, and SC4828 is a putative phosphatase from *Streptomyces coelicolor*.^56^ In addition to structural predictions, we used PaperBLAST^57^ to identify homologs reported in the literature. From this database, we found that Bph has high sequence homology to SA1684, a *Staphylococcus aureus* nucleoside diphosphatase required for expression of secreted proteins and genes regulating virulence and metabolism.^58^ The Tn insertions identified in the primary biofilm screen (Fig. 2b) truncate Bph at the tyrosine in position 101 (Tn1) and near the C-terminus (Tn2).

SC4828 and FomD co-crystallize with phosphate substrates, and purified SA1684 had in vitro phosphatase activity.^58^ The SC4828 residues involved in ligand and metal binding were mostly conserved in Bph (Fig. 6b) despite the low degree of overall sequence similarity between these proteins. Therefore, we hypothesized that Bph is also a phosphatase. To test this prediction, we used a colorimetric in vitro assay to examine phosphatase activity of purified hexahistidine (H6)-tagged Bph protein. Bph-H6 had in vitro phosphatase activity on nucleoside triphosphate, nucleoside diphosphate, and deoxynucleoside phosphate substrates in the presence of Mn^2+^ (Fig. 6c) but not Mg^2+^ or Ca^2+^ (Supplementary Fig. 4). No phosphatase activity was observed with AMP, a monophosphate substrate (Fig. 6c, inset box). We also detected phosphatase activity against ppGpp, the second messenger that mediates cellular responses to a variety of stressors.^59^

To determine whether conserved residues were required for catalytic activity, we generated variants with alanine substitutions at Bph residues predicted to interact with substrate (W70A and Y85A) and coordinate divalent metal cofactors (N87A and D105A). These purified enzymes had no detectable phosphatase activity against ADP, dATP, and ATP in vitro (Fig. 6d). We next asked whether Bph phosphatase activity was required for biofilm formation. We generated complementation plasmids carrying the W70A, Y85A, N87A, and D105A point mutations and transformed them into the Δ*bph* mutant, then measured in vitro biofilm formation after 6 h. Expressing these inactive variants did not restore biofilm production to the amount produced by OG1RF or the wild-type *bph* complementation construct (Fig. 6e). Thus, *Bph* phosphatase activity is required for *E. faecalis* biofilm formation. Because qualitative differences were observed in protein profiles between OG1RF and Δ*bph* strains (Fig. 4b–d), and purified Bph had phosphatase activity in vitro, we predicted that Bph might directly dephosphorylate a cellular protein target. To examine this, we stained whole-cell lysates with the ProQ Diamond phosphoprotein stain, but did not observe any differences in phosphoproteins between OG1RF and Δ*bph* (Supplementary Fig. 5).

## Discussion

**Fig. 7:**
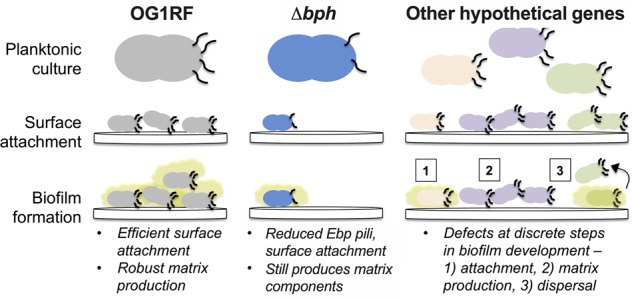
Analysis of discrete steps in biofilm formation as a pathway for characterization of hypothetical gene products. OG1RF (gray, left panel) expresses cell surface factors, such as Ebp pili (black lines) that allow it to attach to substrates and grow as a biofilm in a multi-step process. Biofilm matrix is shown as a yellow shape surrounding cells. Examination of discrete biofilm steps reveals that a strain lacking *bph* (blue, middle panel) has altered expression of Ebp pili and reduced surface attachment, but it can still produce matrix components. Other strains with Tn insertions in hypothetical genes have low-biofilm phenotypes (various colors, right panel). These uncharacterized gene products may be important for discrete biofilm development steps such as (1) surface attachment but not matrix development, (2) elaboration of the biofilm matrix and overall matrix architecture, or (3) controlling the rate of detachment and biofilm dispersal

Nearly a third of the genes annotated in the best-studied bacterial model organisms are poorly characterized.^60,61^ Elucidating the basic function of these gene products and their role in virulence is crucial for understanding microbial pathophysiology and developing next-generation antimicrobial approaches. Here, we took a phenotype-driven approach to study a hypothetical gene involved in surface attachment and early biofilm formation in *E. faecalis* (Fig. 7). Using an arrayed, sequenced Tn library of ~2000 mutations in hypothetical genes and intergenic regions, we identified eight genes where Tn insertions decreased early biofilm formation. Only one (OG1RF_10435, renamed *bph* herein) was required for production of wild type biofilm biomass at both early (*t* = 6 h) and late (*t* = 24 h) time points. This could explain why the other genes were not identified as biofilm determinants in previous genetic screens that examined late biofilm time points.^22,23^ Our findings highlight the importance of understanding the spatiotemporal nature of biofilms and the regulatory pathways that change in biofilms over time.

The primary screen also identified Tn mutants with increased biofilm production relative to OG1RF, suggesting that some *E. faecalis* gene products could negatively regulate biofilm production. Negative biofilm regulators have been found in other organisms like *Vibrio fischeri*.^62^ Although we did not characterize additional high-biofilm or low-biofilm mutants in depth here, another gene of interest is OG1RF_10506. Three Tn insertions in OG1RF_10506 resulted in reduced biofilm formation at 6 h but not 24 h; other than the biofilm defects observed for *bph*-Tn strains, these mutants had the lowest biofilm production at 6 h of strains tested after the primary screen. Structural modeling of OG1RF_10506 revealed homology to polysaccharide deacetylases (Table 1). Interestingly, OG1RF_10506-Tn and OG1RF_10733-Tn mutants also led to a reduction in surface attachment in 96-well plates, although these defects were not as dramatic as the surface attachment defect for *bph* mutants. Our screen also identified numerous biofilm-deficient mutants in which Tn insertions disrupted intergenic regions, suggesting that transcripts derived from these regions may also be important for biofilm development (Supplementary Fig. 1). Future work will elucidate the role of these gene products in biofilm development.

Transitioning from planktonic to biofilm growth requires coordination between numerous gene products to associate with surfaces and elaborate the complex extracellular biofilm matrix. We found that *bph* is important for attachment to microtiter dishes as well as porcine heart valves, suggesting that *bph* could be required for attachment to other host surfaces or cell types. In support of this hypothesis, previous work examining *E. faecalis* gene expression in a subdermal abscess infection model^63^ found that expression of *bph* is increased during early infection but then decreases. Together, this suggests that regulation of *bph* could play a critical role in establishing and maintaining *E. faecalis* infections in vivo.

In *E. faecalis* and other organisms, eDNA is an important matrix component.^15,64,65^ Therefore, it is counterintuitive that planktonic *bph* mutant cells have increased levels of eDNA but reduced attachment and biofilm formation. Autolytic mechanisms for eDNA release have been well-described,^16^ and we previously found that early *E*. faecalis biofilms produce eDNA through a lysis-independent method.^15^ Our results do not suggest that *bph* mutants lyse in culture, but one possibility that could reconcile the disparate biofilm and eDNA phenotypes would be an eDNA release mechanism regulated by the Bph phosphatase that is repressed in wild-type cells during planktonic growth. Another surprising result was that the increased levels of SalB detected in *bph* mutants did not lead to an increase in resistance to cefoxitin, given that SalB mediates cephalosporin resistance in *E. faecalis*.^54^ However, this study suggested that SalB in the extracellular milieu might be inactive, which could explain the increase in extracellular SalB without a resulting increase in antibiotic tolerance.

Bph has a high degree of predicted structural conservation with phosphatases from *Streptomyces* and *S. aureus*. These enzymes target disparate substrates and require varying divalent metal cofactors for activity.^56,58^ Interestingly, *S. aureus* SA1684 exhibited phosphatase activity specifically against diphosphate substrates,^58^ but Bph had in vitro activity against both di- and triphosphates. Bph and SA1684 both contain DUF402 domains that span >90% of the protein length. The DUF402 family is widespread in Firmicutes and Actinobacteria and has been found in archaea like Thermococci and Halobacteria. Many proteins with DUF402 homology have additional domains involved in RNA binding or ribonuclease activities. Given that the DUF402 regions encompass a majority of the coding regions of Bph and SA1684, we propose that DUF402 specifically encodes phosphatase activity, and that the presence of DUF402 alongside other domains is indicative of dual-function proteins.

Although the sequence and in vitro activity of Bph and SA1684 are similar, some aspects of their cellular functions have diverged. Imae et al. found that relative to wild-type, SA1684 mutants had altered protein secretion.^58^ Likewise, we observed differences in secreted proteins between OG1RF and Δ*bph* but also found numerous differences in cytoplasmic proteins. RNAseq of an SA1684 mutant revealed differential expression of virulence genes, genes involved in metabolism (including *pgk*), and numerous hypothetical genes.^58^ Mass spectrometry revealed slightly lower levels of Pgk in our Δ*bph* mutant, but this strain did not have altered growth in medium supplemented with glucose. The arginine deiminase pathway was not differentially regulated in the SA1684 mutant, but we observed lower levels of ArcA protein, as well as an arginine-specific diauxic growth phenotype for the Δ*bph* mutant. Additionally, the Δ*bph* mutant had reduced pili expression, but analogous surface pili have not been identified in *S. aureus*.^66^ Therefore, although Bph and SA1684 are both involved in central metabolic processes, these phosphatases have evolved organism-specific activities.

Expression of surface pili in *E. faecalis* is regulated through several mechanisms. The transcription factor EbpR directly regulates the *ebp* operon as well as itself but does not control expression of the Ace adhesin. AhrC activates expression of both *ebpR* and *ace*,^24^ and the ribonuclease activity of RNase J2 regulates pili and other virulence genes but does not affect *ace*.^41,67^ Here, we showed that a *bph* mutant has reduced expression of *ebpA* and *ebpR* but wild-type levels of Ace and postulate that the overall decrease in pili production in this mutant is through reduced *ebpR* expression. Although it is unlikely that Bph regulates AhrC due to disparate Ace expression in these strains, both gene products are linked to arginine catabolism in addition to pili. AhrC represses expression of the arginine deiminase pathway,^24,32^ whereas we observed reduced levels of ArcA and an arginine-specific growth defect in the absence of *bph*. Arginine modulates adhesion and biofilm formation by some oral bacteria,^68^ but the role of arginine and *E. faecalis* biofilms remains largely unknown. Interestingly, Keogh et al. recently found a link between *E. faecalis* arginine metabolism and polymicrobial biofilms.^69^ l-ornithine, a byproduct of arginine catabolism that is exported by the l-arginine/l-citrulline antiporter ArcD,^51^ is produced by *E. faecalis* biofilms and promotes the development of polymicrobial *E. coli*–*E. faecalis* biofilms by stimulating enterobactin production and iron acquisition by *E. coli*.^69^ Further analysis is needed to elucidate the link between arginine catabolism, pili expression, and biofilm formation and the role of Bph in these pathways.

One unsolved question is the direct in vivo target of Bph phosphatase activity. Bph could act on a small molecule to effect change through nucleotide or second messenger-based signaling pathways, or it could directly dephosphorylate a protein substrate. Interestingly, purified Bph had activity on ppGpp, suggesting that this could be a target in vivo. In cells, increased (p)ppGpp levels lead to the stringent response, a reprogramming event which promotes adaptation to and survival in stress conditions.^59^ (p)ppGpp levels are controlled in *E. faecalis* by RelA and RelQ, and (p)ppGpp-null strains have reduced virulence and survival in macrophages, *C. elegans*, and *G. mellonella*.^70,71^ In vitro, a Δ*relAQ* double mutant formed similar early biofilms to OG1RF with altered biofilm architecture at later time points, possibly due to an increase in detachment of biofilm cells.^72^ A *rel* mutant was defective at attaching to microtiter plates but not ex vivo porcine heart valves, whereas expression of *bph* from a plasmid increased the number of tissue-associated cells in this model.^73^ Although purified Bph had activity on ppGpp, the biofilm phenotypes of this mutant do not phenocopy those of *rel* mutants, so the in vivo relationship between *bph* and (p)ppGpp is unclear.

The mechanistic function of hypothetical proteins in *E. faecalis* and other bacterial organisms is unknown, and studying these gene products requires tractable phenotypes for which they are important. Biofilm development comprises discrete steps that can be precisely targeted experimentally, and controlling the spread of biofilms is of significant interest to the biomedical community. As such, biofilms provide an attractive platform for the analysis of hypothetical protein function. This approach can be expanded to the study of hypothetical proteins in other clinically relevant, biofilm-forming organisms.

## Methods

### Bacterial strains and growth conditions

Bacterial strains used in this study are listed in Table S1. Freezer stocks were stored in 25% glycerol at −80 ^o^C.

Bacteria were cultured in brain–heart infusion (BHI) broth, modified M9 medium with yeast extract and glucose (MM9-YEG),^74^ tryptic soy broth without dextrose (TSB-D) growth medium, or lysogeny broth (LB, for protein overexpression). Agar (1% w/v) was added for solid media, and gelatin (3% w/v) was added where indicated. Antibiotics were used for selection at the following concentrations: chloramphenicol (10 μg/mL), erythromycin (20 μg/mL), fusidic acid (25 μg/mL), gentamicin (100 μg/mL), kanamycin (50 μg/mL), spectinomycin (250 μg/mL), and tetracycline (5 μg/mL). When needed for induction, the pheromone peptide cCF10 was added to a final concentration of 10–50 ng/mL. Antibiotics and gelatin were purchased from Sigma. Growth media components were purchased from Difco.

### Plasmids and oligonucleotides

Plasmids and oligonucleotides used in this study are listed in Table S1. Oligonucleotides were purchased from Invitrogen. PCR was carried out using PfuUltra II Fusion HS DNA polymerase (Agilent Genomics). Restriction enzymes were purchased from New England Biolabs. All constructs were confirmed using Sanger sequencing (Eurofins). Plasmids were transformed into chemically competent *E. coli* for propagation and protein overexpression, and electroporation was used to introduce plasmids into *E. faecalis*.

Overexpression constructs for protein purification were constructed by fusing *bph* variants to the C-terminal hexahistidine (H6) tag encoded on the pET28b+ vector. *bph* lacking the native stop codon was amplified using primers JW109/JW110. Amplicons encoding the Y85A, N87A, and D105A mutations were generated using megaprimers. The first PCR was performed with JW110 and an oligonucleotide containing the desired mutation (Y85A, JW115; N87A, JW116; D105A, JW117) with genomic OG1RF DNA as a template. These products were used in a second reaction to amplify the full-length allele with JW109. The W70A mutation was constructed using overlapping-extension PCR. Two products were amplified from genomic OG1RF DNA using oligo pairs JW109/JW130 and JW114/JW110 and mixed in another reaction with primers JW109/JW110. All full-length alleles were digested with NcoI-HF/XhoI and ligated to pET28b+.

The markerless deletion strain Δ*bph* was generated using allelic exchange and counterselection as described previously,^75^ keeping intact the first and last three codons of the *bph* open-reading frame. The construct for allelic exchange (pJW270) was generated by amplifying genomic regions flanking *bph* with oligos JW133/134 and JW135/136, respectively. PCR products were digested with EcoRI-HF, ligated, then digested with BamHI-HF/SphI-HF and ligated to pCJK218^75^ treated with the same restriction enzymes. Deletion was confirmed by PCR and sequencing.

A tetracycline-resistant derivative of the pheromone-inducible pCIE vector^76^ was constructed by replacing the chloramphenicol resistance cassette with a SpeI/EcoRI-flanked fragment encoding *tetM* to generate pCIE-tet (pJW8). The multi-cloning site of pCIE-tet was replaced with an oligonucleotide cassette (JW29/30) encoding unique BamHI/HincII-SalI/EcoRV/PvuI/NheI restriction enzyme sites, resulting in pCIEtm (pJW76). The full-length *bph* allele and native ribosome-binding site were amplified from genomic DNA using JW163/93. The *bph* point mutants (W70A, Y85A, N87A, D105A) were constructed in the pCIEtm backbone using megaprimers as described above for the pET28b+ constructs, except the reverse primer in the first PCR was JW93, the forward primer in the second PCR was JW163, and the second PCR was done with the wild-type *bph* expression construct as template. Megaprimer PCRs were treated with DpnI to remove template DNA. All PCR products were digested with BamHI-HF/PvuI-HF and ligated to pJW76.

### Arrayed Tn library generation

The arrayed Tn library was derived from an arrayed library generated previously,^30^ which was maintained as frozen stocks in 2D barcoded tubes (Micronic) at the University of Minnesota Genomics Center. Mutants with Tn insertions in genes annotated as hypothetical/unknown (*n* = 1052) or in intergenic regions (*n* = 894) were chosen for inclusion in this library along with 11 control mutants. Individual clones were obtained using an automated XL20 tube handler (BioMicroLab) and inoculated into 96-well plates containing BHI/10% glycerol. Library stock cultures were grown overnight and frozen at −80 ^o^C. The full list of mutants in the library is provided in Supplementary Data 1.

### Biofilm and attachment assays

The biofilm screen of the arrayed Tn library was conducted as previously described with minor modifications.^23,25,77^ All incubations were carried out at 37 ^o^C in a static humidified chamber. A replicating pin (Boekel Scientific) was used to inoculate 96-well plates containing 100 μL fresh TSB-D with the frozen arrayed Tn library strains, and these plates were grown overnight. The next morning, the replicating pin was used to transfer inoculum from overnight cultures to 96-well plates containing 100 μL fresh TSB-D, which were incubated at 37 ^o^C for 6 h. Concurrent control experiments were carried out in a separate plate. A Modulus microplate reader (Turner Biosystems) was used to measure optical density at 600 nm (OD_600_), and planktonic cultures were then removed. Plates were washed three times with double-distilled water using a plate washer (Biotek), dried, and stained with 100 μL safranin (0.01% w/v). Excess safranin was removed by washing the plate three times with double-distilled water. OD_450_ was measured to quantify safranin-stained biofilm material, and biofilm index values were calculated as the ratio of safranin-stained material to cell density (OD_450_/OD_600_) normalized to biofilm produced by OG1RF. All other biofilm assays were performed as described above using overnight cultures grown in TSB-D plus antibiotics and cCF10, and plates were inoculated at a 1:100 dilution.

Mutants with negative biofilm index values were excluded from further analysis. Other OG1RF_10987 and OG1RF_11802 mutants did not have reduced biofilm in this screen, and a general growth defect was observed for the OG1RF_11756 Tn mutant at position 183636. Mutants chosen for further analysis were obtained from the library stock plates and checked for erythromycin sensitivity to ensure loss of the transposase-containing plasmid used in library generation. A third OG1RF_10435-Tn mutant in the arrayed library contains an insertion 82.9% into the gene and had reduced biofilm production in the 6 h biofilm screen (Supplementary Data 2). However, we were unable to isolate an erythromycin-sensitive clone of this mutant, so it was excluded from further analysis.

To measure attachment to microtiter plates, 1 mL aliquots of either overnight cultures (for Tn mutants) or log-phase cultures subcultured from strains grown overnight (for *bph* complementation) were pelleted in a tabletop centrifuge (9615 × *g* for 1 min), washed with 1 volume potassium phosphate-buffered saline (KPBS), resuspended in 100 μL KPBS, and added to a 96-well plate, which was incubated at 37 ^o^C for 1 h. Cell density (OD_600_), attached biomass (OD_450_), and attachment index values (OD_600_/OD_450_) were measured as described for biofilm assays.

Attachment to porcine heart valves was performed as described previously with minor modifications.^36,37^ Pigs were sacrificed for other purposes at the Experimental Surgery Lab at the University of Minnesota (protocol number 1803A35699 approved by the University of Minnesota Institutional Animal Care and Use Committee). Hearts were immediately excised and placed in chilled saline, and valves were removed within 24 h of animal sacrifice. Valve pieces were obtained using a 6 mm punch biopsy tool (Acuderm Inc.) and transferred to a 24-well plate (Corning) containing 2 mL DMEM/5% FBS/gent100. Valves not used immediately were frozen at −80 ^o^C in DMEM/5% FBS/gent100 supplemented with 10% DMSO. Prior to use, valves were incubated overnight in DMEM/5% FBS/gent100 at 37 ^o^C/5% CO_2_, washed 3 × in KPBS, and placed in a 48-well plate with 1 mL DMEM (1 valve piece per well). Wells were inoculated with 5 × 10^7^ bacteria and incubated at 37 ^o^C/5% CO_2_ for 3 h, after which valves were washed 3 × in KPBS and transferred to 15 mL conical tubes containing 5 mL KPBS. Samples were sonicated using a QSonica Q500 sonicator (1.6 mm tip, 20% amplitude, 10 s on/20 s off for a total of 2 min pulse time) to remove tissue-associated bacteria, and aliquots were diluted in KPBS and plated to enumerate CFU/mL. To control for valve size, CFU/mL values were normalized to the weight of each valve piece. We confirm that we complied with all ethical regulations for these experiments.

### CPRG assay

Strains were grown overnight in TSB-D supplemented with erythromycin and were diluted to OD_600_ = 0.05 in fresh medium with erythromycin and 25 μg/mL CPRG. Cultures were incubated statically at 37 ^o^C. Cell density was measured at OD_630_, and CPRG hydrolysis was quantified as the OD_570_ of the culture supernatant after cells were removed by centrifugation (17,000 × *g* for 2 min). The ratio of OD_570/630_ was plotted to evaluate hydrolysis relative to cell growth.

### Gelatinase assays

Cultures were grown overnight in TSB-D and spotted onto TSB-D agar plates supplemented with 3% gelatin (w/v). Plates were incubated at 37 ^o^C overnight for colony growth and then at 4 ^o^C for zone development. Plates were imaged on a ProteinSimple Cell Biosciences FluorChem FC3 imager. Strains were considered gelatinase-positive if an opaque zone developed around the colony.

### eDNA and polysaccharide purification

eDNA was harvested from cultures as previously described^16^ with modifications. Overnight cultures were diluted 1:50 in fresh medium plus antibiotics and cCF10 and grown for 4 h. 1 mL cells were pelleted by centrifugation (17,000 × *g* for 2 min), and supernatants were passed through a 0.22 μm syringe filter. DNA was precipitated using isopropanol and ethanol and resuspended in 10 mM Tris–HCl pH 8.0. Double-stranded DNA was quantified using the Quant-iT PicoGreen dsDNA Assay kit (Thermo Fisher Scientific) according to manufacturer’s instructions. Cultures were diluted and plated to quantify CFU/mL, and values were expressed as fluorescence per CFU/mL.

Polysaccharides were isolated as described previously,^25^ separated using native polyacrylamide gel electrophoresis, and stained using Stains-All (Sigma). Images shown are representative images from three independent experiments. Gels were imaged in grayscale on a BioRad Gel Doc EZ Imager.

### Protein expression and purification

pET28b+ derivatives encoding Bph-H6 variants were transformed into *E. coli* BL21 (DE3) cells and grown in LB/kanamycin. Cultures were grown at 37 ^o^C on an Innova 2300 platform floor shaker (New Brunswick Scientific) at 150 rpm to an OD_600_ of 0.4–0.6, at which point 1.5 mM isopropyl β-d-1-thiogalactopyranoside (IPTG) was added to induce protein expression for 3 h. Cells were harvested by centrifugation (6371 × *g* for 10 min) and resuspended in lysis buffer (20 mM Tris–HCl pH 7.5, 150 mM NaCl, 0.01% Triton X-100, 20 mM imidazole, 100 μg/mL lysozyme, 1 mM PMSF, 10 U/mL DNase). Cells were broken by sonication in an ice bath using a QSonica Q500 sonicator (1.6 mm tip, 40% amplitude, 10 s on/20 s off for a total of 2 min pulse time), and cell debris was pelleted by centrifugation (30,000 × *g* for 15 min). Supernatants were transferred to tubes containing pre-washed nickel NTA resin (Qiagen) and incubated with rotation at 4 ^o^C. The resin was washed 3 × with wash buffer (20 mM Tris–HCl pH 7.5, 150 mM NaCl, 0.01% Triton X-100, 20 mM imidazole), and proteins were eluted (20 mM Tris–HCl pH 7.5, 150 mM NaCl, 250 mM imidazole). Fractions were analyzed for purity using Tris–glycine SDS–PAGE and dialyzed against 2 L buffer containing Tris–HCl pH 7.5, 150 mM NaCl. Proteins were stored at −20 ^o^C in dialysis buffer plus 50% glycerol (v/v).

### Structure predictions and phosphatase assays

The Bph amino acid sequence was used as input for Phyre2^55^ and PaperBLAST.^57^ High-confidence alignments were imported into Pymol.^78^ All structure images were generated using Pymol. For in vitro phosphatase assays, purified Bph-H6 variants (1 μM) were mixed with the indicated substrates (100 μM) in a 96-well plate containing 100 μL reaction buffer (20 mM Tris–HCl pH 7.5, 150 mM NaCl, and 10 mM of the indicated divalent metal cation) per well. Reactions were incubated at 37 ^o^C for 1 h. 4.2% ammonium molybdate in 4 M HCl and 0.045% malachite green (20 μL each) were added to each well followed by 1.4 μL 1% Triton. Absorbance at 640 nm (*A*_640_) was measured using a BioTek Synergy H1 plate reader. *A*_640_ values were normalized to substrate-only controls, and free phosphate was quantified relative to a standard curve. The experiments with Bph-H6 point mutants and divalent cations were performed in parallel, so the control reactions are the same.

### Cell fractionation, protein electrophoresis, and Western blots

To generate protein lysates of *E. faecalis*, overnight cultures were diluted to an OD_600_ of 0.05 into fresh medium and incubated at 37 ^o^C (46 ^o^C for Ace protein expression). At time points indicated in Fig. 4a, the equivalent of 1 mL cells at OD_600_ = 1.0 was pelleted in a tabletop centrifuge (9615 × *g* for 2 min). Western blots were performed with samples harvested at 2 and 4 h, and Pro-Q Diamond staining was done on samples harvested at 4 h. Pellets were resuspended in 100 μL buffer (10 mM Tris (pH 8.0), 1 mM EDTA, 25% sucrose, 15 mg/mL lysozyme) and incubated at 37 ^o^C for 30 min. This suspension was mixed directly with 2× Laemmli sample buffer (BioRad) for whole-cell lysates or centrifuged (17,000 × *g* for 1 min) to separate the pellet (protoplast) from the supernatant (cell wall extract). Secreted proteins in the culture supernatant were precipitated by mixing 1 volume of supernatant with 0.25 volumes of chilled 100% trichloroacetic acid on ice. Precipitated proteins were pelleted in a 4 ^o^C tabletop centrifuge (17,000 × *g* for 10 min) and washed twice with acetone. Pellets were dried and resuspended in urea lysis buffer (8 M urea, 20 mM Tris–HCl pH 7.5, 150 mM NaCl). Proteins were analyzed by SDS–PAGE using 10% Tris–glycine gels. Samples were mixed with 2× Laemmli buffer and heated at 95 ^o^C before loading. Gels were run at 110 V, stained with Coomassie, destained, and imaged on a BioRad Gel Doc EZ Imager.

Phosphoprotein detection was performed with the Pro-Q Diamond Phosphoprotein Gel Stain (ThermoFisher Scientific). Whole-cell lysates were split into untreated and treated fractions, which were treated with chloroform and methanol according to manufacturer’s instructions. Samples were separated by SDS–PAGE, stained, and destained using a modified protocol described previously.^79^ Images were captured using the 600 nm channel on a Licor Odyssey FC Imaging System (LI-COR Biosciences). The gel was then stained with Coomassie and imaged.

For Western blots, proteins were transferred to nitrocellulose membranes (Protran, Sigma-Aldrich) using Towbin buffer (0.05% SDS, 5–20% methanol). Membranes were blocked using 1% milk dissolved in KPBS supplemented with 0.01% Tween-20 (KPBST, pH 7.4). Primary antibodies (1:20,000 anti-Ace and anti-SA80, 1:10,000 anti-EbpC and anti-PrgB) and secondary antibodies (1:20,000 horseradish peroxidase (HRP)-goat anti-rabbit, Invitrogen #65-6120) were diluted in KPBST. Signal was developed using the SuperSignal West Pico chemiluminescent substrate (Thermo Scientific), and images were obtained using the chemiluminescent filter on a Licor Odyssey FC Imaging System. Uncropped Western blots are shown in Supplementary Figs. 6–9. All samples for a given Western blot were derived from the same experiment and processed in parallel.

### Mass spectrometry

Overnight cultures were diluted in fresh medium and cultured for 4 h, at which point cell lysates were prepared for gel electrophoresis as described above. Gel slices were excised from Coomassie-stained Tris–glycine SDS–PAGE gels with a razor blade and were submitted to the University of Minnesota College of Biological Sciences Center for Mass Spectrometry and Proteomics for in-gel trypsin digest and proteomic analysis by LC/MS–MS on an LTQ Orbitrap Velos mass spectrometer. All MS/MS samples were analyzed using Sequest (XCorr Only) (Thermo Scientific, version IseNode in Proteome Discoverer 2.2.0.388) to search *E. faecalis* OG1RF proteins assuming the digestion enzyme trypsin with a fragment ion mass tolerance of 0.100 Da and a parent ion tolerance of 50 ppm. Scaffold (version Scaffold_4.8.9, Proteome Software Inc.) was used to validate MS/MS-based peptide (>77.0% probability, FDR < 1.0% by scaffold local FDR) and protein (>97.0% probability, FDR < 1.0%, at least two identified peptides) identifications. Protein probabilities were assigned by the Protein Prophet algorithm.^80^ Percent total spectra were compared between OG1RF and Δ*bph* samples to identify the most abundant differentially expressed proteins.

### Growth curves and antibiotic sensitivity assays

Growth in media supplemented with glucose or arginine was performed using a semisynthetic medium described previously.^52^ Strains were grown in TSB-D, pelleted in a tabletop centrifuge (9615 × *g* for 2 min), and washed in semisynthetic medium. Cells were diluted to a starting OD_600_ of 0.05 in 200 μL base medium or medium supplemented with 1% glucose or 1% arginine in a 96-well plate, which was sealed with Microseal B PCR plate sealing film (Bio-Rad). OD_600_ measurements were taken at 15 min intervals for 15 h in a BioTek Synergy H1 plate reader. For antibiotic sensitivity assays, strains were grown in TSB-D and adjusted to OD_600_ = 0.05 in 200 μL of two-fold dilutions of antibiotics (highest concentrations: cefoxitin, 512 μg/mL; oxacillin, 64 μg/mL) in a 96-well plate. 15 h growth curves were carried out as described above, and the ending OD_600_ value for each condition was divided by the OD_600_ of the untreated culture.

### Conjugation

Overnight cultures of donors and recipients were grown in TSB-D or TSB-D/tet, diluted 1:10 in fresh TSB-D without antibiotics, and incubated at 37 ^o^C for 1 h. Cells were mixed at a 1:9 donor:recipient ratio in a microfuge tube. At indicated time points, aliquots were removed, diluted in KPBS supplemented with 2 mM EDTA, and plated on selective antibiotic agar plates.

### Microscopy

*E. faecalis* biofilms were grown on Aclar fluoropolymer substrates essentially as described previously.^15,24^ Overnight cultures were diluted 1:50 in TSB-D medium supplemented with 50 ng/mL cCF10 and cultured for 6 h in a 24-well polystyrene plate (Corning) at 100 rpm on a MaxQ 2000 tabletop shaker (Thermo Scientific). Cells were stained with 10 μg/mL Hoechst 33342 (Molecular Probes/Thermo Fisher Scientific), and images were captured with a Zeiss AX10 wide-field microscope using Zen2 (blue edition) software with a ×20 0.8-numerical-aperture objective. Images were imported as grayscale and false-colored (LookUp Table: cyan) using Fiji^81^ and cropped to 500 × 500 pixels using GIMP (https://www.gimp.org/).

### Statistical analysis

For experiments where numerical values are presented, individual data points from three independent biological replicates are shown. Error bars indicate standard error of the mean. Statistical significance was assessed using analysis of variance (ANOVA) with corrections for multiple comparisons by controlling the false discovery rate. Statistical analysis was performed using GraphPad Prism. For assays where qualitative images are shown, experiments were performed in triplicate, and representative images are shown.

## Supplementary information

Supplemental Material

data set 1

data set 2

## Data Availability

The files generated from mass spectrometry are available through Figshare at 10.6084/m9.figshare.9627257. All other data generated or analyzed during this study are included in this published article and its supplementary information files.

## Supplementary information

**Supplementary information** accompanies the paper on the *npj Biofilms and Microbiomes* website (10.1038/s41522-019-0099-0).
