# Supplementary material for: Exploiting biofilm phenotypes for functional characterization of hypothetical genes in Enterococcus faecalis
Source: NPJ Biofilms Microbiomes. 2019 Sep 19;5:23. doi: 10.1038/s41522-019-0099-0 (PMC6753144; doi:10.1038/s41522-019-0099-0)
Supplement: Supplementary file 1 — Supplemental Material [file 41522_2019_99_MOESM1_ESM.pdf]

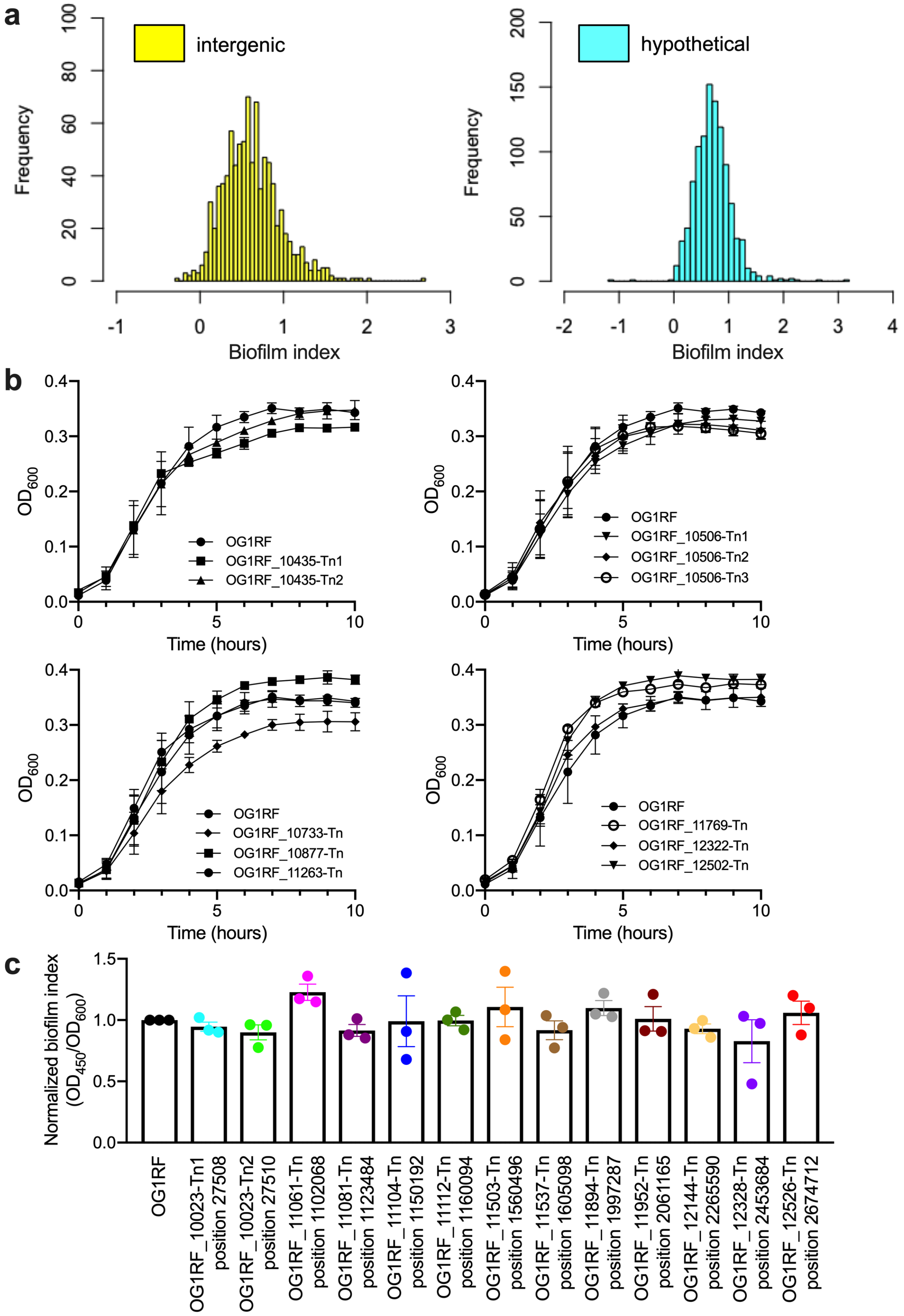

a
intergenic
Frequency
Biofilm index
hypothetical
Frequency
Biofilm index
b
OD600
Time (hours)
OG1RF
OG1RF_10435-Tn1
OG1RF_10435-Tn2
OG1RF
OG1RF_10506-Tn1
OG1RF_10506-Tn2
OG1RF_10506-Tn3
OG1RF
OG1RF_10733-Tn
OG1RF_10877-Tn
OG1RF_11263-Tn
OG1RF
OG1RF_11769-Tn
OG1RF_12322-Tn
OG1RF_12502-Tn
c
Normalized biofilm index (OD450/OD600)
OG1RF
OG1RF_10023-Tn1 position 27508
OG1RF_10023-Tn2 position 27510
OG1RF_11061-Tn position 1102068
OG1RF_11081-Tn position 1123484
OG1RF_11104-Tn position 1150192
OG1RF_11112-Tn position 1160094
OG1RF_11503-Tn position 1560496
OG1RF_11537-Tn position 1605098
OG1RF_11894-Tn position 1997287
OG1RF_11952-Tn position 2061165
OG1RF_12144-Tn position 2265590
OG1RF_12328-Tn position 2453684
OG1RF_12526-Tn position 2674712

Supplementary Figure 1. Biofilm screen of arrayed transposon library and *in vitro* attachment of selected low-biofilm mutants. a) Distribution of biofilm index values relative to OG1RF (biofilm = 1.0) obtained in the arrayed Tn library screen. Insertions in intergenic regions are represented by yellow bars, and insertions in hypothetical genes are represented by cyan bars. **b)** Growth curves of putative low-biofilm mutants selected from initial Tn library screen. Mutants were grown in a 96-well plate in tryptic soy broth without added dextrose. Values are the average of three independent replicates with independent error of the mean. **c)** Biofilm production at 6 hr was measured for Tn mutants with high levels of biofilm in the primary screen. Each data point represents a biological replicate. The nucleotide position of each Tn insertion is indicated in the x-axis labels.

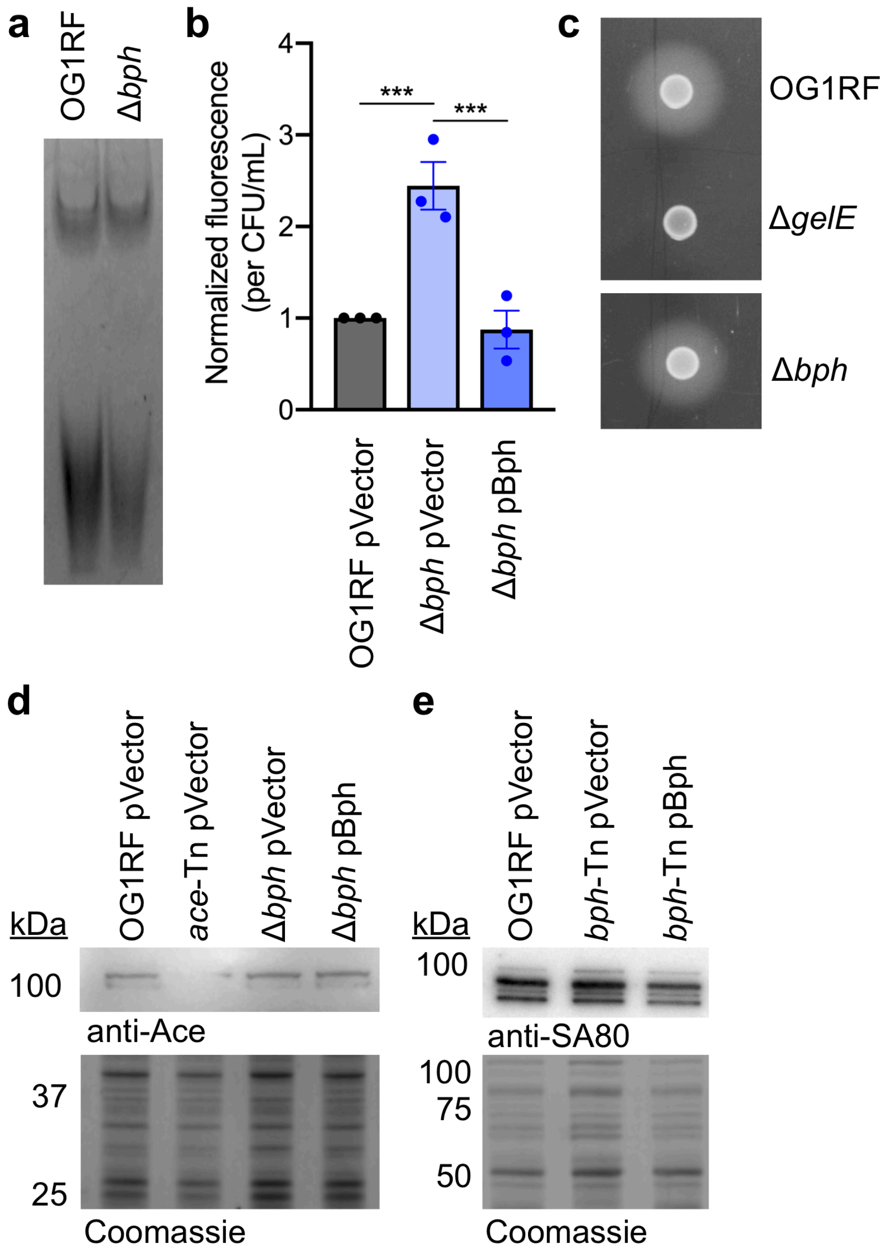

Supplementary Figure 2. *bph* mutations do not affect general biofilm determinants or surface proteins. a) Polysaccharides harvested from cells in mid-logarithmic phase were separated on a native gel. **b)** Extracellular DNA was quantified using PicoGreen and normalized to CFU/mL of the cell culture. Values are presented relative to parental OG1RF. Each data point represents a biological replicate. Statistical significance was determined by one-way ANOVA (***q<0.001). **c)** Gelatinase activity was measured using agar plates supplemented with 3% gelatin. Hazy zones surrounding colonies are indicative of gelatinase activity. **d)** Ace levels were detected in whole-cell lysates via Western blot with an anti-Ace antibody (top panel). **e)** SA80 was detected in the cell wall fraction via Western blot with an anti-SA80 antibody (top panel). For **d** and **e**, Coomassie-stained load controls are shown (bottom panels).

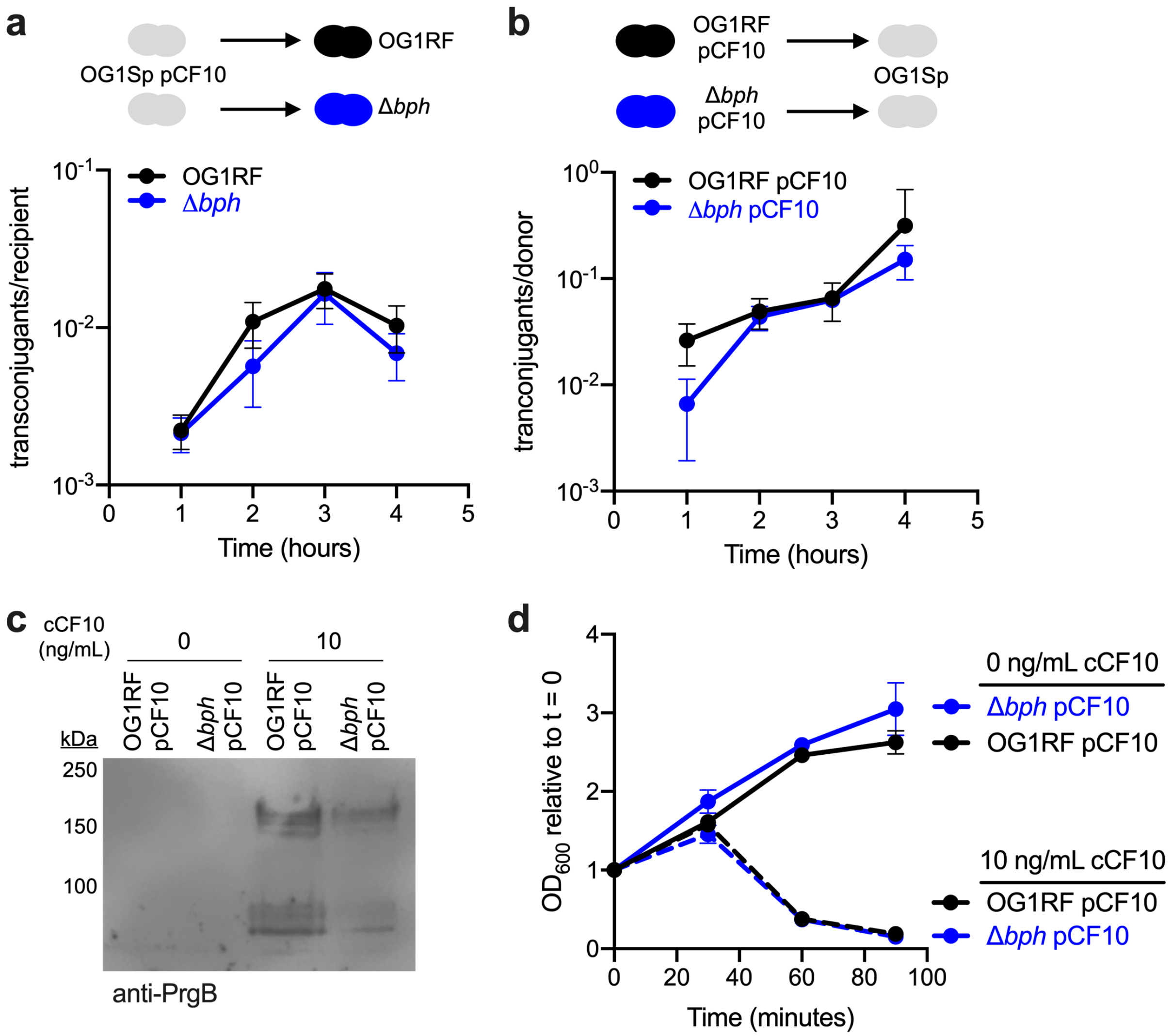

Supplementary Figure 3. Effect of *bph* on conjugation and transfer of pCF10. Transfer of pCF10 between cells was measured using **a)** OG1RF and Δ*bph* as recipients and **b)** OG1RF and Δ*bph* carrying pCF10 as donor strains. Cells were grown separately for 1 hr and mixed at a 9:1 recipient:donor ratio. At indicated timepoints, transconjugants, donors, and recipients were selected on antibiotic agar plates and enumerated. Data points represent three biological replicates, and error bars represent standard error of the mean. **c)** Whole-cell lysates were harvested 1 hr after induction with 10 ng/mL cCF10. Proteins were separated using SDS-PAGE and transferred to nitrocellulose, and aggregation substance was detected using an anti-PrgB antibody. **d)** Aggregation of OG1RF and *Δbph* carrying pCF10 after induction with exogenous cCF10 (10 ng/mL) was measured by tracking the OD_{600} at the top of individual cultures.

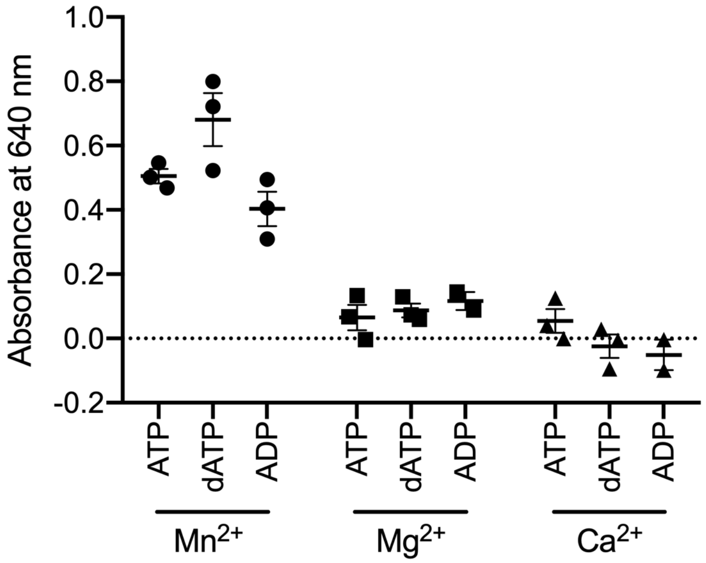

Supplementary Figure 4. Bph requires manganese for phosphatase activity. Purified Bph-H6 was mixed with the indicated substrates in reaction buffer supplemented with various divalent cations. Free phosphate (measured by absorbance at 640 nm) was detected when manganese was used as the divalent cation, but not magnesium or calcium. Each data point represents a biological replicate. Error bars represent standard error of the mean.

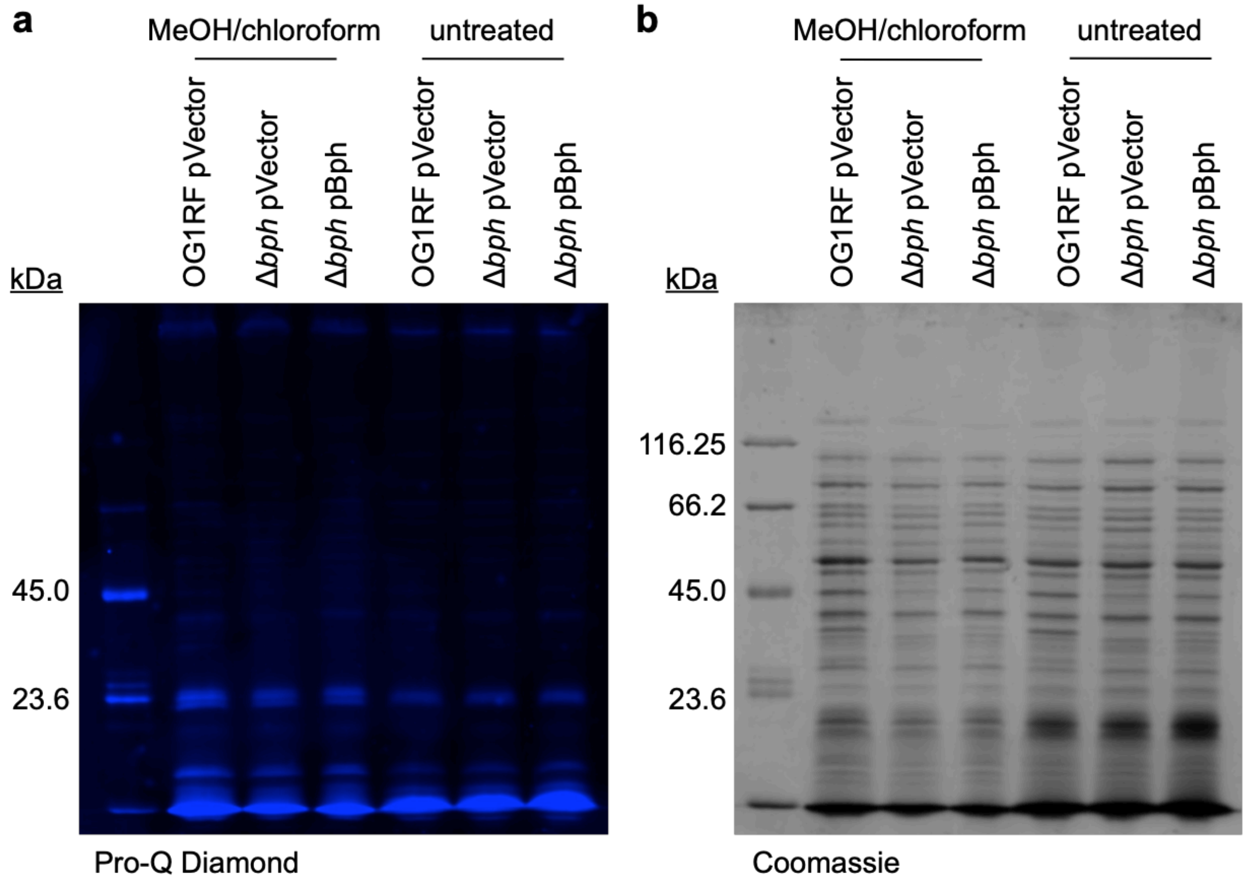

Supplementary Figure 5. Effect of Bph on phosphoprotein content. Whole-cell lysates were prepared from the indicated strains and separated by SDS-PAGE. **a)** The gel was incubated in ProQ Diamond phosphoprotein stain and imaged. Phosphoproteins are visualized as blue bands. **b)** The gel from panel **a** was incubated in Coomassie stain and imaged.

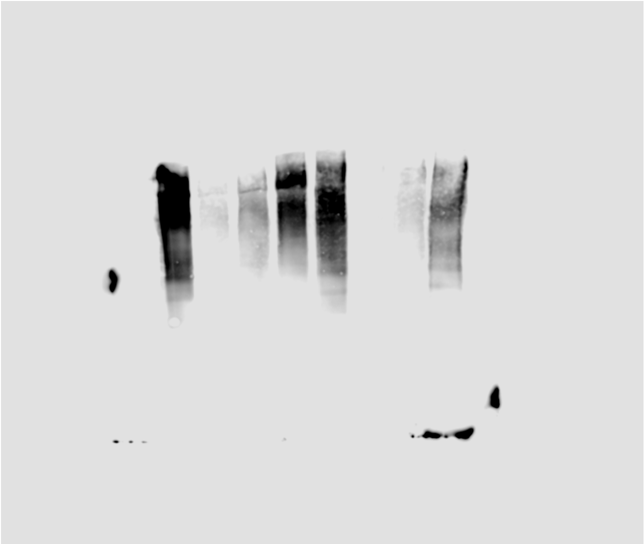

Supplementary Figure 6. Uncropped anti-EbpC Western blot.

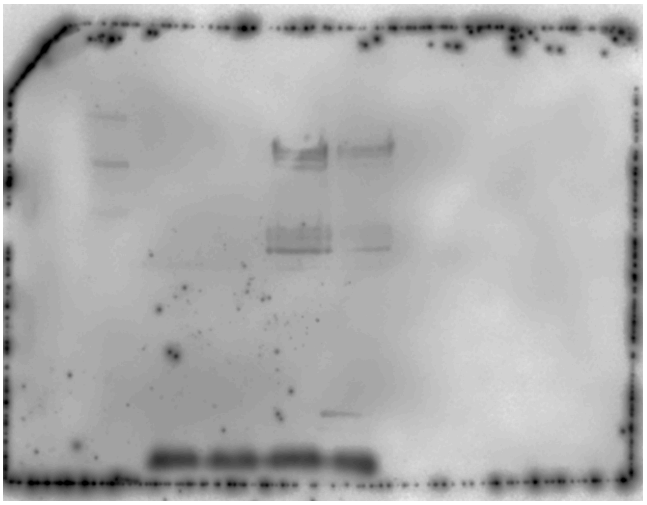

Supplementary Figure 7. Uncropped anti-PrgB Western blot.

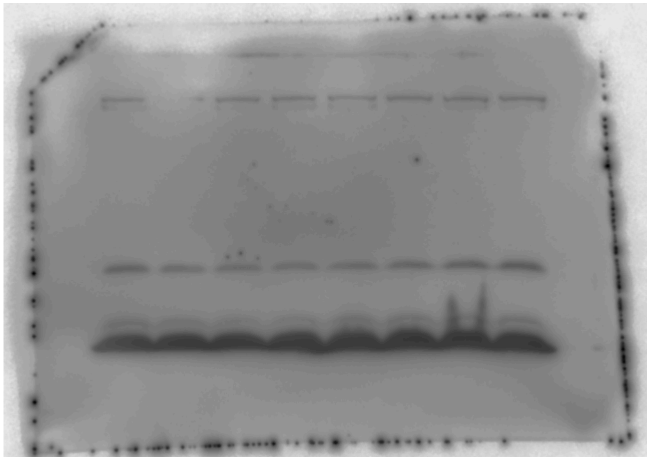

Supplementary Figure 8. Uncropped anti-Ace Western blot.

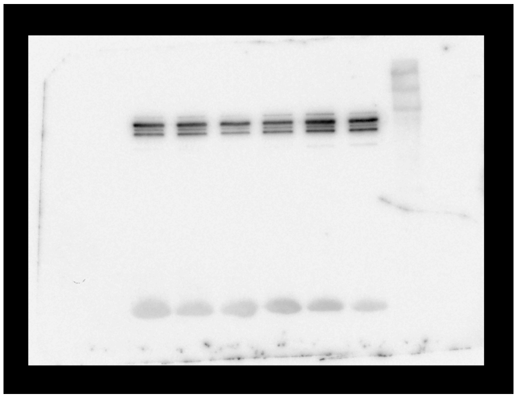

Supplementary Figure 9. Uncropped anti-SA80 Western blot.

Strain	***Description***	***Relevant Characteristics***	***Source***
	Enterococcus faecalis OG1RF	Parent strain, Rif^R, Fus^R	[1]
JW271	*Enterococcus faecalis* OG1RF Δ10435 (Δ*bapA*)	Markerless deletion of OG1RF_10435, Rif^R, Fus^R	This study
	Enterococcus faecalis OG1RF Δ*ahrC*	Markerless deletion of *ahrC* (OG1RF_10717), Rif^R, Fus^R	[2]
	Escherichia coli DH5α	Laboratory K-12 cloning strain	Fisher Scientific
	Escherichia coli BL21 (DE3)	Laboratory strain for T7 expression	New England Biolabs
	Plasmids	***Relevant Characteristics***	***Source***
pJW8	pCIE-tet	Pheromone-inducible pCIE vector with tetracycline resistance cassette, Tet^R	This study
pJW76	pCIE-tet-MCS (pCIEtm)	pCIE-tet with modified multicloning site (BamHI/HincII-SalI/EcoRV/PvuI/NheI), Tet^R	This study
pJW250	pCIEtm::OG1RF_10435	Contains wild-type OG1RF_10435 (*bapA*) sequence with native ribosome-binding site, Tet^R	This study
	pCJK205	P_{23}::*lacZ* constitutive reporter plasmid derived from pMSP3535, Erm^R	[3]
	pCJK218	Temperature-sensitive plasmid for allelic exchange and *pheS* counterselection, Cm^R	[4]
	pCJK218::OG1RF_10435-del	Allelic exchange plasmid containing genomic regions flanking OG1RF_10435, Cm^R	This study
	pET28b+	Plasmid vector carrying N-	Novagen

		terminal and C-terminal His6 tags and an N-terminal thrombin-T7 tag, Kan^R	
pJW204	pET28b+::OG1RF_10435-H6	OG1RF_10435 coding sequence fused to C-terminal His6 tag	This study
pJW246	pET28b+::OG1RF_10435-H6 W70A	OG1RF_10435 coding sequence with W70A mutation fused to C-terminal His6 tag	This study
pJW233	pET28b+::OG1RF_10435-H6 Y85A	OG1RF_10435 coding sequence with Y85A mutation fused to C-terminal His6 tag	This study
pJW237	pET28b+::OG1RF_10435-H6 N87A	OG1RF_10435 coding sequence with N87A mutation fused to C-terminal His6 tag	This study
pJW235	pET28b+::OG1RF_10435-H6 D105A	OG1RF_10435 coding sequence with D105A mutation fused to C-terminal His6 tag	This study
pJW336	pCIEtm::OG1RF_10435 W70A	pJW250 (OG1RF_10435 complementation vector) with W70A mutation, Tet^R	This study
pJW337	pCIEtm::OG1RF_10435 Y85A	pJW250 (OG1RF_10435 complementation vector) with Y85A mutation, Tet^R	This study
pJW338	pCIEtm::OG1RF_10435 N87A	pJW250 (OG1RF_10435 complementation vector) with N87A mutation, Tet^R	This study
pJW339	pCIEtm::OG1RF_10435 D105A	pJW250 (OG1RF_10435 complementation vector) with D105A mutation, Tet^R	This study
	pTCV-LacSpec	pTCV-LacSpec, vector for promoter fusions, Spec^R	[2]
	pTCV-LacSpec::prom-*ebpR*	Promoter fusion P*ebpR*(- 87) in pTCV-LacSpec, Spec^R	[2]
	pTCV-LacSpec::prom-*ebpA*	Promoter fusion P*ebpA*(- 100) in pTCV-LacSpec, Spec^R	[2]
	Oligonucleotides	***Sequence***	***Source***
JW54	pCIE-tetM-MCS	5' - AAA GTC GAC GAT ATC CGA TCG GCT AGC CCT AAA GAA GTA ACC ATG TAT TAT G - 3'	This study
JW59	10435-fwd	5' - TTT AAG TGA ACT CAA TGT ACG C - 3'	This study
JW60	10435-rev	5' - ACA AGT CAC CGC TGT AGG C - 3'	This study

JW93	10435-Pvu-rev	5' - TTT CGA TCG CTA TTT TTT TGA TAG CTG TTG ATA ACG - 3'	This study
JW109	10435-Nco-fwd	5' - ATA CCA TGG GAA TTC CTA AAG AAG G - 3'	This study
JW110	10435-Nhe-Xho-rev	5' - ATA CTC GAG GCT AGC TTT TTT TGA TAG CTG TTG ATA ACG -3'	This study
JW114	10435-W70A-fwd	5' - CAC AAG AAA TAT GCG TTC AAT ATA ATA GC - 3'	This study
JW115	10435-Y85A-fwd	5' - GGG TTT CGT ATG CCT GCA ATC TGG CTT CG - 3'	This study
JW116	10435-N87A-fwd	5' - GGG TTT CGT ATT ACT GCG CTC TGG CTT CGC C - 3'	This study
JW117	10435-D105A-fwd	5' - GTA TAT TGA TTA TGC TTT AGA TAT CAA GG - 3'	This study
JW130	10435-W70A-rev	5' - GCT ATT ATA TTG AAC GCA TAT TTC TTG TG - 3'	This study
JW133	10435-up-fwd-Bam	5'- GTT GGA TCC AGA ATA TGG CC -3'	This study
JW134	10435-up-rev-Eco	5' - TAG GAA TTC CCA TAA TAC CCC - 3'	This study
JW135	10435-down-fwd-Eco	5' - ATA GAA TTC CTA AAT AGC CGA TGT AAA CAG C - 3'	This study
JW136	10435-down-rev-Sph	5' - ACT GCA TGC TAG CTG ATC TGC C - 3'	This study
JW163	10435-fwd-Bam	5' - TTT GGA TCC AAG TGA ACT CAA TGT ACG C - 3'	This study

Supplementary Table 1. Bacterial strains, plasmids, and oligonucleotides used in this study. Cm^R, chloramphenicol; Fus^R, fusidic acid; Rif^R, rifampicin; Tet^R, tetracycline; $Spec^R$, spectinomycin. Restriction enzyme sites in oligonucleotides are included in the name or in parentheses.

1 Dunny, G., Funk, C. & Adsit, J. Direct stimulation of the transfer of antibiotic resistance by sex pheromones in Streptococcus faecalis. *Plasmid* **6**, 270-278 (1981).

2 Manias, D. A. & Dunny, G. M. Expression of Adhesive Pili and the Collagen-Binding Adhesin Ace Is Activated by ArgR Family Transcription Factors in Enterococcus faecalis. *J Bacteriol* **200**, doi:10.1128/JB.00269-18 (2018).

3 Djorić, D. & Kristich, C. J. Oxidative stress enhances cephalosporin resistance of Enterococcus faecalis through activation of a two-component signaling system. *Antimicrob Agents Chemother* **59**, 159-169, doi:10.1128/AAC.03984-14 (2015).

4 Vesić, D. & Kristich, C. J. A Rex family transcriptional repressor influences H2O2 accumulation by Enterococcus faecalis. *J Bacteriol* **195**, 1815-1824, doi:10.1128/JB.02135-12 (2013).
